# Effects of supplementation of chromium histidinate on glucose, lipid metabolism and oxidative stress in cats

**DOI:** 10.1111/jpn.13023

**Published:** 2018-11-22

**Authors:** Nina Machac, Gulsah Kaya Karasu, Nurhan Sahin, Cemal Orhan, Kazim Sahin, Christine Iben

**Affiliations:** ^1^ Department of Farm Animals and Veterinary Public Health, Institute of Animal Nutrition and Functional Plant Compounds University of Veterinary Medicine Vienna Vienna Austria; ^2^ Department of Animal Nutrition, Faculty of Veterinary Medicine Firat University Elazig Turkey

**Keywords:** cats, chromium histidinate, glucose metabolism, glucose tolerance, lipid metabolism, oxidative stress

## Abstract

In recent years, two meta‐analyses of chromium (Cr) supplementation have shown beneficial effects on glucose metabolism. Chromium histidinate (CrHis) reduces serum glucose levels in rats fed a high‐fat diet but no study has been conducted on cats until now. The aim of this study was to examine the effects of CrHis on glucose and lipid metabolism in cats. To challenge the glucose metabolism, 16 cats were fed a high‐carbohydrate high‐fat diet for three months. One group (*n* = 8) received 800 ug CrHis per day for two months, while the other group (*n* = 8) served as control group. An oral glucose tolerance test was conducted, blood samples were taken, and biochemical parameters and oxidative stress were measured. CrHis serum levels were significantly increased (*p* = 0.027) in the treatment group, while fructosamine levels were significantly lower (*p* = 0.029) in the control group. In both groups, glucose (*p* < 0.01), b‐hydroxy‐butyrate (*p* = 0.024) and 8‐hydroxy‐deoxyguanosine (*p* = 0.028) levels decreased significantly and cholesterol levels increased significantly (*p* < 0.01). In conclusion, CrHis did not improve glucose or lipid metabolism and did not affect oxidative stress in healthy cats.

## 1 INTRODUCTION

Diabetes mellitus is a growing problem worldwide, not only in humans, but also in pet animals. Treatment of diabetes mellitus often is challenging, especially in cats. Therefore, a food supplement that improves insulin sensitivity and facilitates therapy would be valuable.

A poor intake of Cr per se, an increased loss of Cr via urine because of high intake of simple sugars and an increased requirement due to insulin resistance leads to a Cr deficiency (Anderson, 1998; Anderson, Bryden, Polansky, & Ryson, 1990; Bahijiri, 2000).

There is evidence that Cr supplementation is only successful in individuals with a Cr deficiency or if there a glucose intolerance has already been diagnosed (Anderson, 1998; Bahijiri, 2000; Sreejayan, Dong, Kandadi, Yang, & Ren, 2008).

According to the societies for nutrition in Germany (Deutsche Gesellschaft für Ernährung), Austria (Österreichische Gesellschaft für Ernährung) and Switzerland (Schweizerische Gesellschaft für Ernährung), an appropriate intake for adult human beings is between 30 and 100 µg daily (DGE, 2017). No references are available for cats.

Numerous studies have been conducted to determine whether supplementation of Cr can improve glucose and lipid metabolism, but their results are inconsistent. A meta‐analysis of 14 randomised controlled trials on adults with diabetes mellitus type 2 indicates only a small benefit of Cr. In a subgroup analysis, brewer's yeast showed a significant decrease in fasting plasma glucose, whereas other Cr complexes did not alter HbA1C or fasting plasma glucose (Yin & Phung, 2015).

In a study by Appleton, Rand, Sunvold, and Priest (2002), 32 healthy, non‐obese cats were separated into a control group and three treatment groups. They received diets with different chromium tripicolinate amounts (150, 300 and 600 ppb, respectively) over 6 weeks. Cats receiving more than 300 ppb Cr had decreased glucose concentrations, and their area under the glucose–concentration–time curve was lower than that of the controls. Cats fed 600 ppb chromium tripicolinate (CrPic) had significantly decreased fasting glucose concentrations. These results indicate an improved glucose metabolism after the supplementation of CrPic in healthy, non‐obese cats.

Bahijiri (2000) reports not only improved glucose control but also reduced triglycerides and increased HDL cholesterol levels in healthy humans. Liver triglyceride levels were found to be reduced in obese mice fed Cr‐d‐phenylalanine (Sreejayan et al., 2008).

In individuals with diabetes mellitus, oxidative stress is increased (Song et al., 2007). F2‐isopostranes are the gold standard to measure oxidative stress, malondialdehyde (MDA) is generated by lipid peroxidation, and 8‐hydroxy‐deoxyguanosine (8‐OH‐2dG) is a marker for an oxidative attack on DNA (Stephens, Khanolkar, & Bain, 2009).

Beneficial effects of Cr supplementation on glucose and lipid metabolism are observed in different species, but there is not much literature about the effects of Cr in cats. There are only a few studies available for CrHis in humans and rodents; these suggest a better absorption than other Cr complexes. Since there are no data of the effects of CrHis in cats, the aim of our study is to find potential positive effects of CrHis on glucose and lipid metabolism, as well as improvement of the oxidative stress status in cats.

We hypothesised that supplementation of 800 µg of CrHis improves glucose metabolism, lipid metabolism and oxidative stress status even in healthy cats.

## 2 MATERIALS AND METHODS

All procedures have been approved by the ethical committee of the University of Veterinary Medicine Vienna and authorized by the Austrian Federal Ministry of Science, Research and Economy (Reference Number BMWF‐68.205/0054‐II/10b/2010).

### 2.1 Animals

Altogether, 16 cats (seven males, nine females; 11 neutered, five intact) participated in this study. The breed was either European shorthair or Maine coon × European shorthair. Mean age was 2.82 ± 1.5 years (1 year–5 years). Their initial body weight was 4.24 ± 0.97 kg (2.85–6.06 kg). All cats had body condition score 5–6/9 (Laflamme, 1997). The body weight was controlled weekly and body weight change was restricted to a maximum of 5% of the initial body weight.

One cat became ill with a mild form of feline upper respiratory tract disease and was treated with marbofloxacin, acetylcysteine and a gentamycin eye ointment for four days. Another cat was treated with low doses of prednisolone. These cats were not excluded from the trial, and all treatments stopped two weeks before chromium supplementation.

### 2.2 Diet

Cats were fed a mixture of a dry and a wet commercial diet for adult cats, enriched with lard (76.93% wet diet (composition: meat and animal derivatives (40%), cereals, vegetable protein extracts, minerals, sugar), 15.38% dry diet (composition: cereals, meat and animal derivatives (29%, i.a. 4% beef), oils and fats, vegetable protein extract, derivates of vegetable origin, minerals, vegetables (4% carrots, 4% peas)), 7.69% lard). The energy and nutrient content of the dry and wet diets (proximate analyses) as well as of the mixed diet (calculated from the analysed values) is shown in Table 1. The amount of food was calculated according to the energy requirements of the cats (0.42 MJ ME/kg body weight^0.67^). The chromium content of the diet was not analysed. This mixed diet was fed to all cats throughout the whole period of three months. Cats were fed twice daily and had ad libitum access to water. Food intake was recorded daily by weighing food bowls before and after feeding.

**Table 1:** Energy content^a^ (MJ ME/100 g dry substance) and crude nutrient content (% dry substance) of the dry and the wet diet (proximate analyses) and the mixed diet^b^ (calculated)

	Wet diet	Dry diet	Mixed diet^b^
MJ ME	1.849	1.703	2.131
Dry substance	18.14	93.25	35.99
Crude protein	49.10	34.71	32.88
Crude fat	22.56	12.75	35.20
Crude fibre	0.76	1.61	0.94
Crude ash	14.60	8.02	8.86
N‐free extr.	12.95	42.90	22.12

Notes

^a^calculated using the formula of NRC (2006);

^b^76.93% wet diet, 15.38% dry diet, 7.69% lard.

### 2.3 Experimental design

During the first period of 28 days, all cats were fed a fat‐enriched diet, as described above. After this period, cats were randomised into two groups, each consisting of eight cats. The detailed group composition is shown in Table 2. One group was the control group, while the other group received 800 µg of CrHis (202 µg of Cr; Nutrition 21, Inc., Purchase, NY, USA) additionally for eight weeks. CrHis was mixed into the food. None of the cats refused the food.

**Table 2:** Cats in chromium and control group

	Chromium	Control
Age (years)	2.41 ± 1.66	3.23 ± 1.29
Weight (kg)	3.88 ± 0.97	4.61 ± 0.87
Female	2	1
Male	1	1
Female neutered	3	3
Male neutered	2	3

An oral glucose tolerance test (OGTT) was conducted, and blood samples were collected at the start of the experiment (day 0) and afterwards every four weeks. Whole blood count, blood chemistry (glucose, fructosamine, insulin, cholesterol, triglyceride and β‐hydroxy‐butyrate) and Cr levels were analysed. To monitor oxidative cell damage, 8‐isoprostane, 8‐hydroxy‐2‐deoxyguanosine (8‐OH‐2dg) and malondialdehyde (MDA) serum levels were determined.

**Figure 1:**
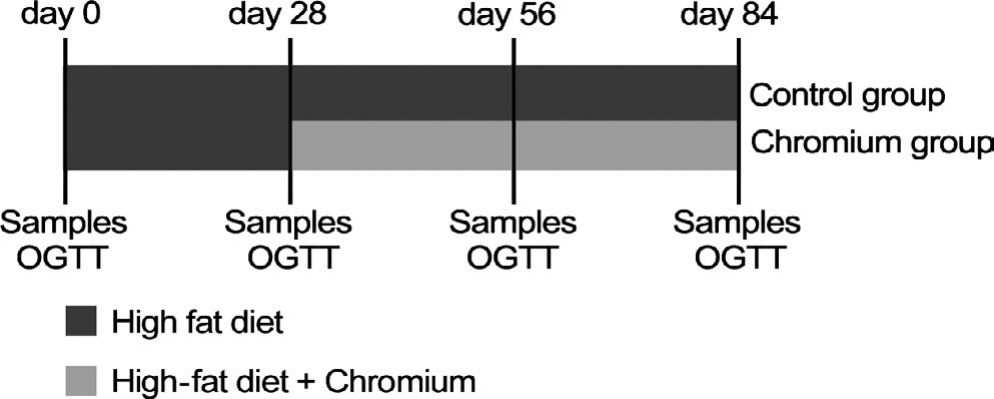
Experimental design: timeline

Timeline is shown in Figure 1.

### 2.4 OGTT

The oral glucose tolerance test was performed four times—first before starting the diet on day 0, second after four weeks (day 28), just before starting the Cr supplementation and then every four weeks (day 56 and day 84). Food bowls were removed 12 hr before the glucose tolerance test to ensure that cats had fasted before OGTT.

Cats received 2 g of glucose (C_6_H_12_O_6_ * H_2_O) per kg body weight per os. Blood samples were collected from *V. cephalica* at 0 and 30 min after glucose intake. Capillary blood glucose concentrations were measured with a validated portable blood glucose meter (Freestyle Freedom glucometer, Abbott Diabetes Care Inc, Alameda, USA) after puncturing of the ear at 0, 15, 30, 60, 120 and 180 min after glucose administration. The glucometer was validated by Zeugswetter, Benesch, and Pagitz (2007).

Time of glucose elimination from blood (*k*_gluc_) and glucose half‐life (*t*_1/2_) were calculated by the linear regression analysis of the semi‐algorithmic plot of glucose concentration versus time between maximum glucose concentration and baseline after glucose administration. Area under the glucose curve (AUC_gluc_) for the entire 180 min period was calculated using the trapezoidal method.

### 2.5 Sample handling and laboratory analysis

Haematology, blood chemistry and insulin levels were analysed immediately at the University of Veterinary Medicine Vienna, Austria. Cr, MDA, 8‐OH‐2dG and 8‐isoprostane were analysed at the Firat University, Turkey.

Blood samples were collected in the morning before the OGTT. Therefore, 6 ml of blood was collected from the *V. cephalica* into adequate tubes (Vacuette LH lithium‐heparin 2 ml for blood chemistry, Vacuette K2E K2EDTA 2 ml for haematology and Vacuette Z Serum Sep Clot Activator 2.5 ml for T4, Cr, MDA, 8‐OH‐2dG and 8‐isoprostane; Greiner Bio one GmbH, Kremsmünster, Austria). The samples were centrifuged immediately (2,400 rpm, 10 min). Sera were separated, filled in 500‐µl vials and stored at −80°C.

Fully automated methods were used at the Central Laboratory, University of Veterinary Medicine, Vienna, for analysing haematology and blood chemistry. Haematology was performed on an ADVIA 2120^TM^ (Siemens Healthcare Diagnostics Inc., Tarrytown, USA) with a software adapted specifically for veterinary species. It is a laser‐based flow cytometer for haematological analysis. For blood chemistry analysis, Cobas 501c (Roche Diagnostics, Vienna, Austria), a fully selective auto‐analyser for clinical chemistry, was used. Methods were applied according to the manufacturer's recommendations (glucose: enzymatic hexokinasetest; fructosamine: nitroblue tetrazolin assay; β‐hydroxy‐butyrate: D‐3 OHB esterase assay; triglyceride: Wahlefeld method; cholesterol: CHOD‐PAP method; creatinine: enzymatic caliometric creatininase/quinone imine test, total protein: biuret method, albumin: photometric test using bromocresol green; alkaline phosphatase: kinetic‐photometric test according to IFCC standard; glutamate pyruvate transaminase, glutamate oxaloacetate transaminase: UV test according to IFCC standard modified; glutamate dehydrogenase: kinetic photometric test according to Szasz/Persijn). A two‐level quality control was performed before each analytical run to ensure appropriate analyser function.

Insulin was measured by a commercially available ELISA test kit (Mercodia Feline Insulin ELISA, Mercodia, Uppsala, Sweden).

Cr concentrations were measured by graphite furnace atomic absorption spectrophotometer with an autosampler (Perkin Elmer Analyst 800 with Zeeman background correction, The Perkin Elmer Corporation, Norwalk, USA) (Chromium Lumina Hollow Cathode Lamp, wavelength 357.9 nm, THGA graphite tube).

Serum was diluted 1:1 with 0.2% nitric acid. The negative standard was nitric acid; for Cr standard, 13 ml of 65% nitric acid was diluted in 36 ml distilled water. After this, 0.5 g of Mg(NO_3_)2.6 H_2_O was added. Then, 2.5 ml of this standard Cr dilution (20 ppb Cr) was diluted with 2.5 ml of 0.2% nitric acid. Samples of 1.5 ml were put into the autosampler. Samples were analysed at least twice, and the mean value was calculated.

For measuring 8‐isoprostane and 8‐OH‐2dg, an ELISA kit was used (Oxis International, Inc., Foster City, CA; Bioxytech 8‐OhdG‐EIA, Assay, 8‐Isoprostane Assay) according to the manufacturer's protocols.

MDA serum concentrations were quantified by a high‐performance liquid chromatograph (Shimadzu, LC‐20 AD pump, SIL‐20 A autosampler, UV‐vis SPD‐10 AVP detector, inertsil ODS‐3 C18 column [250 × 4.6 mm, 5 µm], CTO‐10ASVP column oven, DGU‐20A5 degasser) (Shimadzu Scientific Instruments, Columbia, USA). Mobile phase was 30 mM of KH_2_PO_4_ methanol (8.5 + 17.5, vol/vol %, pH 3.6), and flow rate was 1.2 ml/min. Injection volume was 20 µl. The chromatograph was monitored at 250 nm.

### 2.6 Statistical analysis

Statistical analysis was performed with SPSS 17 Inc. (Statistical Package for the Social Sciences, IBM Corporation, Armonk, USA).

To assess differences between groups at each instance of sample collection, a one‐way analysis of variance (ANOVA) was used. Significant differences were tested using the paired *t* test for normally distributed data and Wilcoxon rank test for non‐normally distributed data. Significance was achieved at *p* < 0.05.

## 3 RESULTS

All results are shown in Table 3.

**Table 3:** Laboratory measurements before the trial (day 0) and at days 28, 56 and 84

	Group	Day 0	Day 28	Day 56	Day 84
Cr (mg/L)	Chromium^b^	1.68 ± 0.99	2.34 ± 0.98†	3.88 ± 1.25† ^,^ *	2.76 ± 0.58† ^,^ *
	Control^b^	1.24 ± 0.70	1.64 ± 0.52	1.86 ± 0.62	1.24 ± 0.45
Gluc_0_ (mg/dl)	Chromium^e^	76.50 ± 8.65	85.13 ± 12.25	83.75 ± 7.89	77.75 ± 9.11
	Control^e^	76.50 ± 11.43	75.00 ± 11.54	78.13 ± 8.72	74.88 ± 8.34
Gluc_30_ (mg/dl)	Chromium^e^	161.25 ± 43.44	131.0 ± 20.65	137.63 ± 23.77	117.38 ± 30.99†
	Control^e^	158.5 ± 24.64	140.5 ± 19.60	146.13 ± 31.61	134.63 ± 31.61†
Ins_0_ min (ng/L)	Chromium^e^	—	188.52 ± 59.67	81.33 ± 50.42†	292.06 ± 217.77
	Control^e^	—	132.98 ± 42.31	27.90 ± 15.89†	259.23 ± 306.33
Ins_30_ min (ng/L)	Chromium^e^	—	414.14 ± 130.76	293.83 ± 230.57†	416.58 ± 251.07
	Control^e^	—	333.25 ± 103.26	185.73 ± 111. 85†	369.02 ± 250.21
Fruc (µmol/L)	Chromium^a^	271.00 ± 29.02	269.88 ± 24.67*	256.63 ± 25.02†	253.38 ± 17.95†
	Control^a^	269.25 ± 18.203	242.75 ± 14.94† ^,^ *	246.13 ± 20.04†	241.13 ± 19.4†
β‐HB (mmol/L)	Chromium^a^	0.08 ± 0.05	0.06 ± 0.03	0.06 ± 0.01	0.05 ± 0.02†
	Control^a^	0.12 ± 0.14	0.06 ± 0.02	0.06 ± 0.03	0.05 ± 0.02†
Tri (mg/dl)	Chromium^a^	28.13 ± 7.88	29.00 ± 6.35	35.5 ± 17.73	727.00 ± 7.48
	Control^a^	30.00 ± 9.83	25.63 ± 7.60	25.25 ± 5.26	26.38 ± 6.30
Chol (mg/dl)	Chromium^a^	197.88 ± 48.94	187.63 ± 36.06	201.75 ± 31.87	215.63 ± 22.48†
	Control^a^	183.38 ± 10.85	189.88 ± 35.79	203.5 ± 44.91	214.88 ± 50.14†
MDA (nmol/L)	Chromium^b^	1.50 ± 0.63	2.00 ± 0.98	1.64 ± 0.22	1.73 ± 0.37
	Control^b^	1.93 ± 0.70	2.09 ± 0.40	2.22 ± 0.84	2.22 ± 0.45
8‐OH‐2dg (nmol/L)	Chromium^e^	29.04 ± 2.89	27.82 ± 1.98	25.5 ± 2.05†	26.21 ± 2.54†
	Control^c^	30.01 ± 3.54	26.22 ± 29.91	26.85 ± 1.91†	26.26 ± 2.84†
8‐iso (nmol/L)	Chromium^d^	35.84 ± 4.55	37.31 ± 13.80	31.64 ± 5.66	31.48 ± 3.95
	Control^d^	34.22 ± 7.87	35.32 ± 3.59	39.23 ± 5.87	38.70 ± 7.60

Notes

8‐iso: 8‐isoprostane; 8‐OH‐2dg: 8‐hydroxydeoxyguanosine; Chol: cholesterol; Cr: chromium; Fruc: fructosamine; Gluc_0_: fasting glucose; Gluc_30_: glucose 30 min after glucose challenge; Ins_0_: fasting insulin; Ins_30_: insulin 30 min after glucose challenge; MDA: malondialdehyde; Tri: triglyceride; β‐HB: β‐hydroxy‐butyrate.

Data are given in arithmetic mean ± *SD*.

a *n* = 8.

b *n* = 7.

c *n* = 6.

d *n* = 5.

e *n* = 4.

*Significant difference between groups, *p* < 0.05.

†Significant change over time, *p* < 0.05.

### 3.1 Food intake, body weight, side effects/health profiles

All 16 cats completed the study. All cats maintained good appetite and no problems with Cr administration occurred. Food intake of the cat with a mild form of feline upper respiratory tract disease was decreased on two days. Food intake of the cat treated with prednisolone did not change. The body weight change of the cats did not exceed 5% for any cat.

General health profile (blood haematology and biochemistry), tested monthly, remained unchanged throughout the three‐month trial in all cats, and no side effects of CrHis were observed.

### 3.2 Chromium serum levels

**Figure 2:**
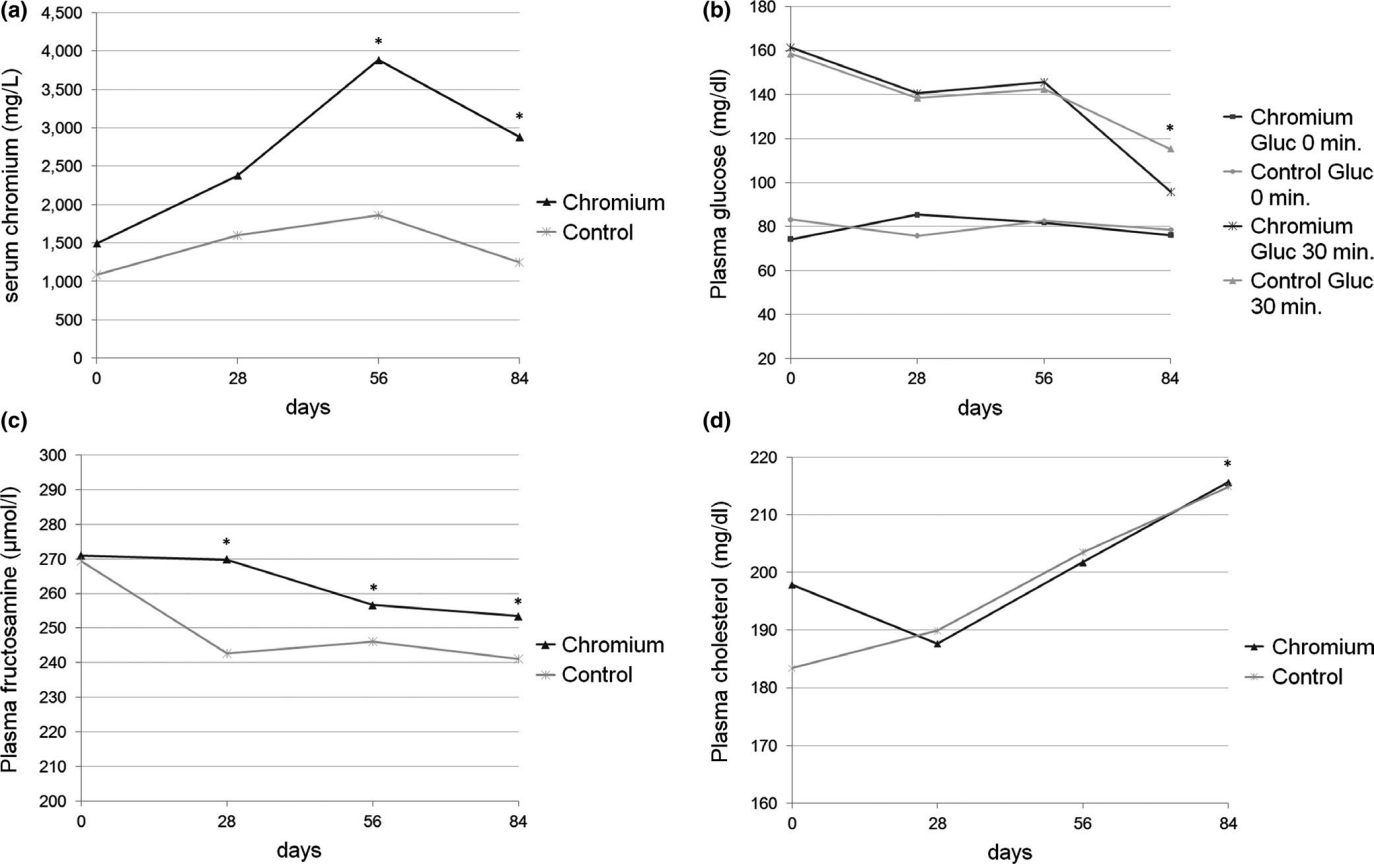
(a) Serum Cr levels, significant group effect *p* < 0.01. (b) Plasma glucose levels before (Gluc 0) and 30 min after (Gluc 30) glucose challenge. (c) Plasma fructosamine levels, significant group effect *p* = 0.029. (d) Plasma cholesterol levels; *significant difference to baseline *p* < 0.05

There was no significant difference between groups before starting the Cr supplementation. The Cr serum levels increased significantly in the group receiving the CrHis (*p* < 0.01). Cr levels tended to differ between groups at day 28 (*p* = 0.098) and differed significantly between groups at day 56 (*p* = 0.02) and at day 84 (*p* < 0.01). Serum Cr levels increased significantly after Cr supplementation in the CrHis group, indicating that Cr supplementation was successful. Cr concentrations are shown in Figure 2.

### 3.3 Glucose metabolism

Fasting glucose concentrations measured from *V. cephalica* were significantly different from glucose concentrations 30 min after glucose load, indicating that orally administered glucose was well‐absorbed (*p* < 0.01).

Glucose levels were significantly lower at the end of the trial (*p* = 0.007). Plasma glucose levels 30 min after glucose load changed significantly more throughout the trial than fasting glucose concentrations (*p* = 0.022). No effect of Cr treatment was detected (Figure 2).

Fructosamine plasma levels decreased significantly (*p* < 0.01); there was a significant difference between groups (*p* = 0.029), as shown in Figure 2.

Insulin plasma levels differed significantly before and after glucose challenging (*p* < 0.01) in both groups.

Fasting insulin concentrations and insulin concentrations 30 min after glucose load decreased significantly at day 56 (*p* < 0.05) and tended to decrease at day 84 (*p* = 0.057), but without group effect. Cr had no effect on plasma insulin concentrations.

Beta‐hydroxy‐butyrate levels significantly decreased (*p* = 0.024). Both the CrHis group and the control group were affected similarly, indicating no effect of Cr supplementation.

OGTT did not indicate any difference in glucose metabolism. Rate of glucose elimination (*k*_gluc_), glucose half‐life (*t*_½_) and area under the glucose curve (AUC_gluc_) were not different from pre‐treatment levels.

### 3.4 Lipid metabolism

There was no effect of CrHis treatment on triglycerides. Triglyceride levels did not change significantly between measurements either.

Cholesterol levels increased significantly (*p* < 0.01) in all animals without group effect (Figure 1).

### 3.5 Oxidative stress

8‐OH‐2dg levels were significantly lower at the end of the trial (*p* = 0.028) in both groups. There was no significant change in MDA levels. 8‐isoprostane levels tended to be lower in the Cr group than in the control group (*p* = 0.081).

## 4 DISCUSSION

Cr has been discussed as having a beneficial effect on glucose and lipid metabolism since the 1950s (Mertz, 1998). The Cr absorption in the intestine is quite low—between 0.4% and 3% depending on references (Anderson, Polansky, & Bryden, 2004; Mertz, 1998; Rhodes et al., 2010). To improve Cr absorption, we chose CrHis, which was better absorbed than CrPic as shown in a study by Anderson et al. (2004). In the present study, CrHis was absorbed very well, as shown by increased Cr plasma levels in the CrHis group but not in the control group following the Cr supplementation.

Nonetheless, we could not detect any effect of the CrHis treatment. This corresponds to a meta‐analysis of 14 randomised controlled trials, in that only Cr yeast and no other Cr complex improved fasting blood glucose. However, CrHis was not included in this meta‐analysis (Yin & Phung, 2015).

Effects of Cr may be dose‐dependent, as shown in a study by Appleton et al. (2002). Therefore, we chose a relatively high dose of 800 µg CrHis, per cat which was higher than in several other studies in cats, dogs and humans (Bahijiri, 2000; Cohn, Dodam, McCaw, & Tate, 1999; Schachter, Nelson, & Kirk, 2001). However, dosage of CrPic in the study by Appleton et al. (2002) could not be directly compared to the dosage of CrHis in our study as CrPic contains 12.43% elemental Cr, while CrHis contains 25.22% elemental Cr (Sahin et al., 2017).

Regardless of this high dose, we did not observe any side effects of Cr treatment. Two months of Cr supplementation seem to be enough, as shown in numerous previous studies (Amoikon et al., 1995; Appleton et al., 2002; Bahijiri, 2000). Nevertheless, in this study, CrHis supplementation affected neither fasting glucose levels nor OGTT. Because there was a four‐week period of feeding the specified diet before chromium supplementation, it can be assumed that any significant differences over time may be affected by the different food. Before starting this trial, cats received wet food only, which contained less fat and carbohydrates (N‐free extracts) and more protein than the food given during the trial period.

Since fasting glucose is maintained by a lot of mechanisms, it is not surprising that fasting glucose levels are often unaffected by Cr supplementation (Amoikon et al., 1995; Bahijiri, 2000; Kleefstra et al., 2007).

It is suggested that Cr supplementation only works in individuals with a lack of Cr or with an impaired glucose tolerance (Anderson, 1998; BahijiriMira, Mufti, & Ajabnoor, 2000; Mertz & Schwarz, 1959). In the present study, we used healthy cats that were of normal weight and probably had a normal Cr status. To induce Cr deficiency, we fed a diet with a high amount of carbohydrate and added fat (lard) over three months because of reports that an increased intake of carbohydrate and fat leads to an increased loss of Cr in human beings and could lead to Cr deficiency (Bahijiri, 2000; Bahijiri et al., 2000). A high intake of carbohydrates and fat has been observed to lead to reduced liver Cr concentrations and increased insulin and glucose serum levels in rats (Tuzcu et al., 2011). There is no literature available to indicate whether a high carbohydrate or high fat intake induces a Cr deficiency in cats. A typical diet of feral cats contains only 2% Nfe (Verbrugghe & Hesta, 2017). As obligate carnivores, cats have a different glucose metabolism as compared to humans, dogs or rodents. The liver of cats shows no activity of glucokinase, and they have comparatively longer blood glucose elimination times (Kienzle, 1994; NRC, 2006).

Cr is necessary for the action of chromodulin that enhances the activation of insulin receptor in insulin‐sensitive tissue in an insulin‐dependent manner and therefore leads to increased insulin‐sensitivity (Vincent, 2000). Chromium supplementation to animals that were rendered insulin‐resistant by either genetic or nutritional methods indicates that chromium potentiates the actions of insulin and upregulates cellular glucose uptake (Hua, Clark, Ren, & Sreejayan, 2012).

It can be assumed that the action of chromium in cats is the same as described above. Due to the fact that cats are in a constant state of gluconeogenesis and enzymes of gluconeogenesis are much higher than that in dogs (NRC, 2006; Tanaka et al., 2005), the effect of chromium could be less effective compared with other animals or humans. Besides that, cats have low activities of the intestinal disaccharidases sucrase and lactase (Kienzle, 1993) as well as pancreatic amylase activity; nevertheless, cats are able to digest carbohydrates properly (Morris, Trudell, & Pencovic, 1977).

Although triglyceride levels were unaffected in our study, plasma cholesterol levels increased following high‐fat diet, indicating that lipid metabolism was impaired. Also Appleton et al. (2002) did not observe an impact of chromium supplementation on cholesterol level in cats. The authors suggested that the 6‐week duration may have been too short. In humans, a decrease in cholesterol occurs after 12 weeks of chromium supplementation but was not evident at 6 weeks (Wang, Fox, Stoecker, Menendez, & Chan, 1989). Obviously, the duration in our study was too short as well.

In the present study, plasma glucose levels 30 min after glucose load decreased significantly and insulin levels tended to decrease, indicating an improved glucose tolerance. But CrHis group and control group were affected similarly. This indicates that CrHis had no effect on glucose tolerance or insulin response in healthy cats.

Fructosamine levels differed between the groups. Fructosamine concentrations decreased in the control group after one month of the diet but stayed constant in the Cr group. However, CrHis started for both groups after the first month, so the decrease could not be caused by CrHis.

In our study, β‐hydroxy‐butyrate plasma levels decreased significantly, but without group effect. Beta‐hydroxy‐butyrate plasma levels correlate well with the glucose plasma concentrations and fructosamine plasma concentrations in diabetic cats and are markers for glucose intolerance as well (Zeugswetter, Handl, Iben, & Schwendenwein, 2010). Mean β‐hydroxy‐butyrate plasma concentrations in our study did not exceed reference values. Hyperglycaemia and hyperlipidaemia are associated with increased oxidative stress (Song et al., 2007; Stephens et al., 2009). 8‐OH‐2dg decreased significantly in both groups independent of Cr supplementation. 8‐hydroxy‐deoxyguanosine is a marker for oxidation of DNA and is used to measure whole‐body oxidative stress (Frijhoff et al., 2015).

Although 8‐isoprostane is the gold standard to measure oxidative stress, the correlation with the metabolic syndrome is poor and other diseases such as chronic renal insufficiency or cystic fibrosis affect 8‐isoprostane levels to a much greater extent (van't Ervea et al., 2017). 8‐isoprostane levels in our study tended to decrease in the CrHis group after the second month implying reduced oxidative stress as a result of CrHis supplementation.

## 5 CONCLUSION

This study reveals that CrHis seems to be absorbed well by cats, and this compound can be recommended for Cr supplementation in suspicious cases. There was no effect of additional Cr supplementation to commercial diets on glucose or fat metabolism in healthy cats. The oxidative stress may be influenced by trend even in healthy cats, as the 8‐isoprostane levels were observed to be lower in the CrHis group.
